# Supplementary material for: Examining the Potential of Vitamin C Supplementation in Tissue-Engineered Equine Superficial Digital Flexor Tendon Constructs
Source: Int J Mol Sci. 2023 Dec 4;24(23):17098. doi: 10.3390/ijms242317098 (PMC10707379; doi:10.3390/ijms242317098)
Supplement: Supplementary file 1 [file ijms-24-17098-s001.zip › Figures.pdf]

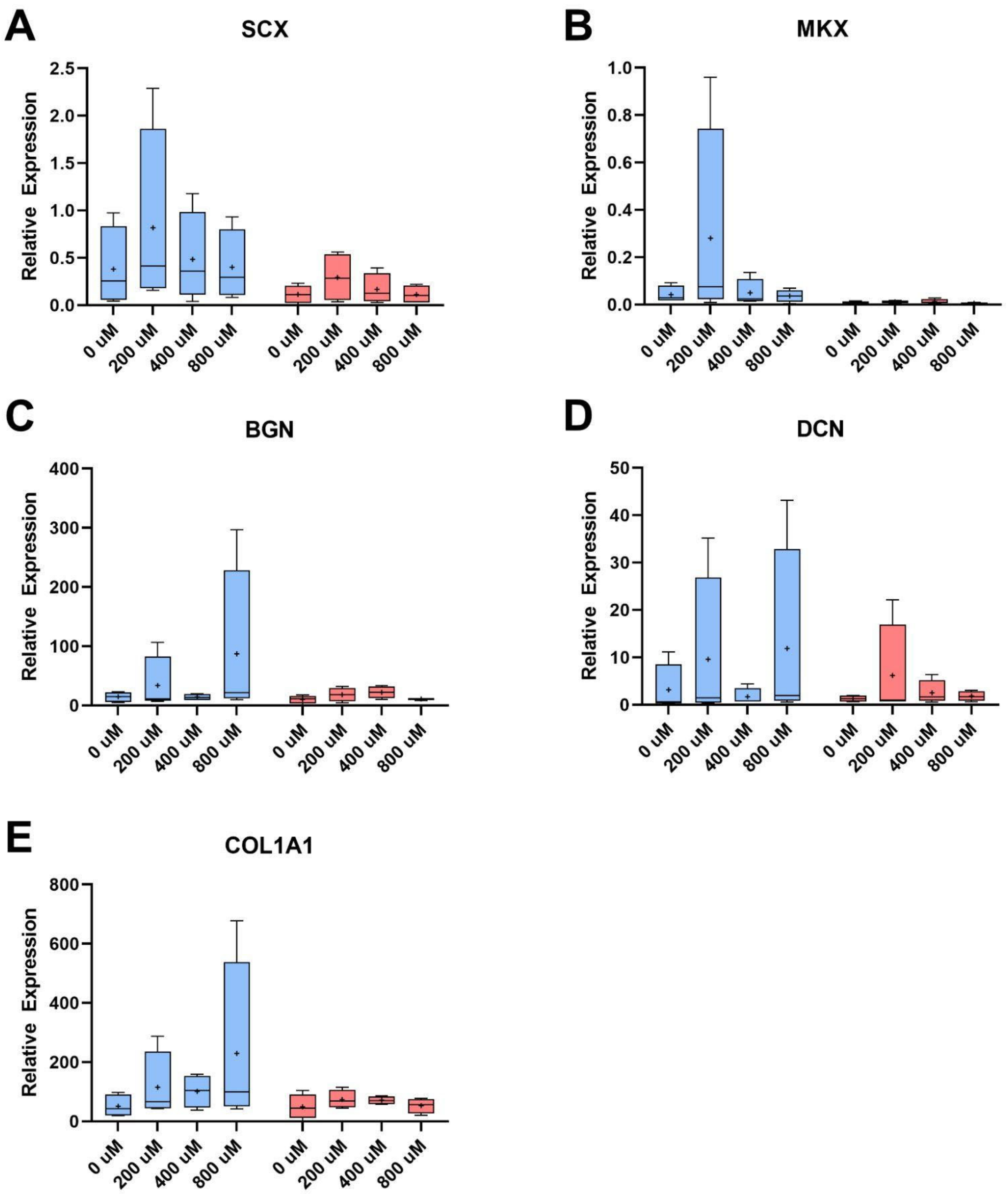

Figure S1. RT-qPCR of genes associated with tendon differentiation and matrix assembly for TP and PERI cells in tendon constructs supplemented with Vitamin C. Gene expression was examined for *SCX* **(A)**, *MKX* **(B)**, *BGN* **(C)**, *DCN* **(D)**, and *COL1A1* **(E)** for TP (blue) and PERI (red) cell-derived constructs supplemented with Vitamin C. RT-qPCR statistical analyses performed to examine expression of tendon markers were two-tailed non-parametric tests with Dunn's multiple test correction (n=4).

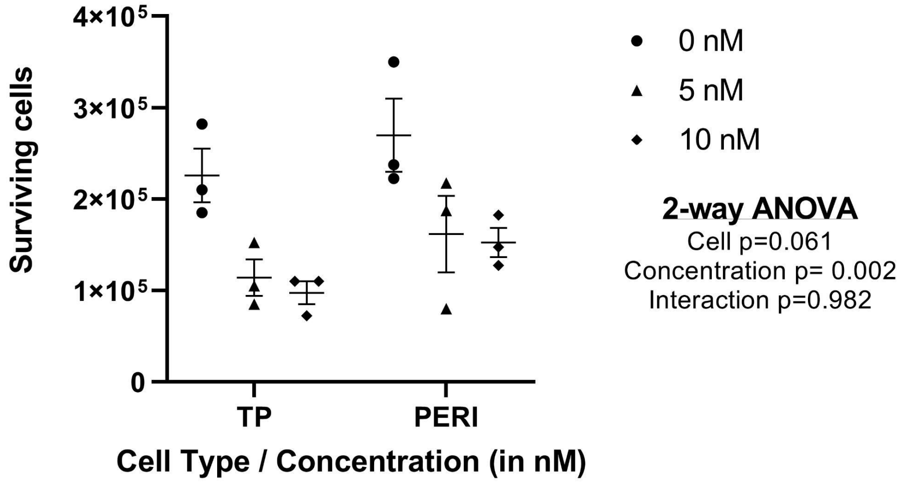

Figure S2. TP and PERI cells were challenged with dexamethasone for 3 days. Concentrations of 5 nM and 10 nM performed equally as well, so 10 nM was selected for the dexamethasone challenge and Vitamin C rescue experiments (n=3).

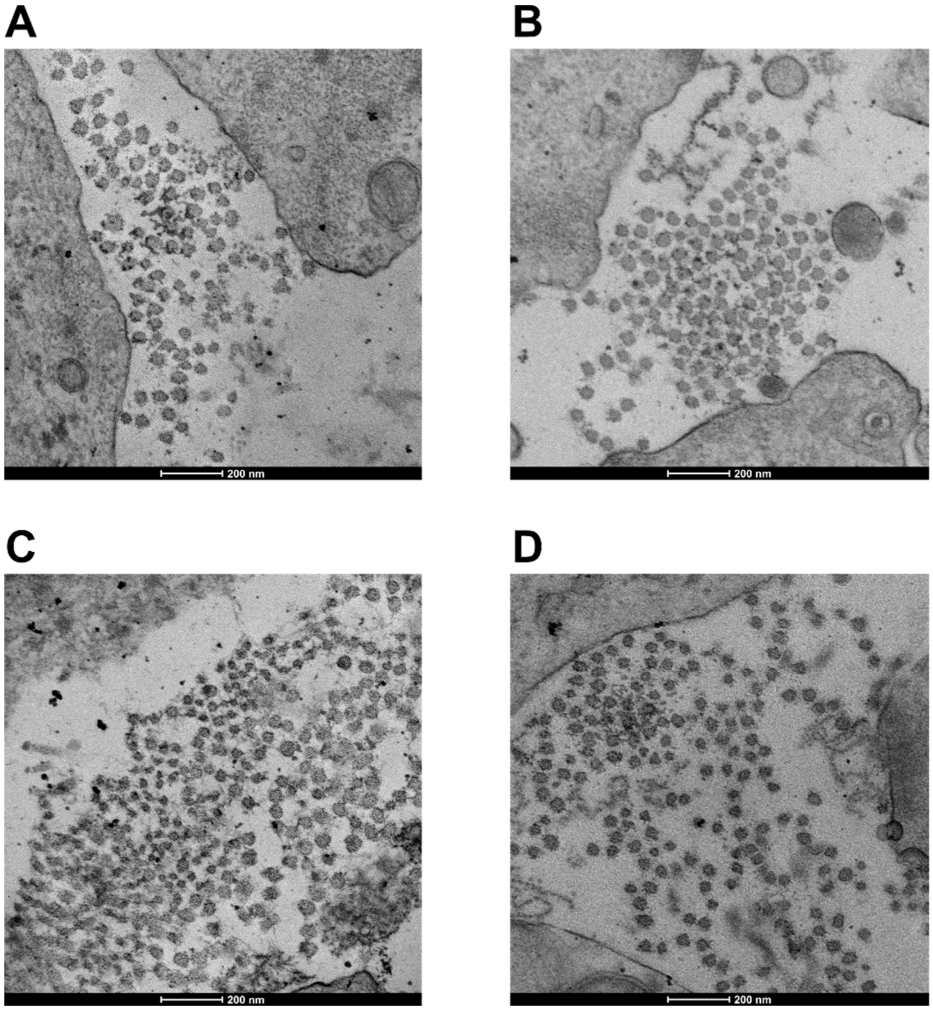

Figure S3. Representative TEM images. Fibril diameters were measured using transmission electron microscopy. For each fibril seen in each image, its diameter was measure in ImageJ. Images shown are representative. Depicted are images from TP cell-derived constructs supplemented with 200 µM **(A)** or 400 µM **(B)** vitamin C, as well as from PERI cell-derived constructs supplemented with 200 µM **(C)** or 800 µM **(D)** vitamin C.

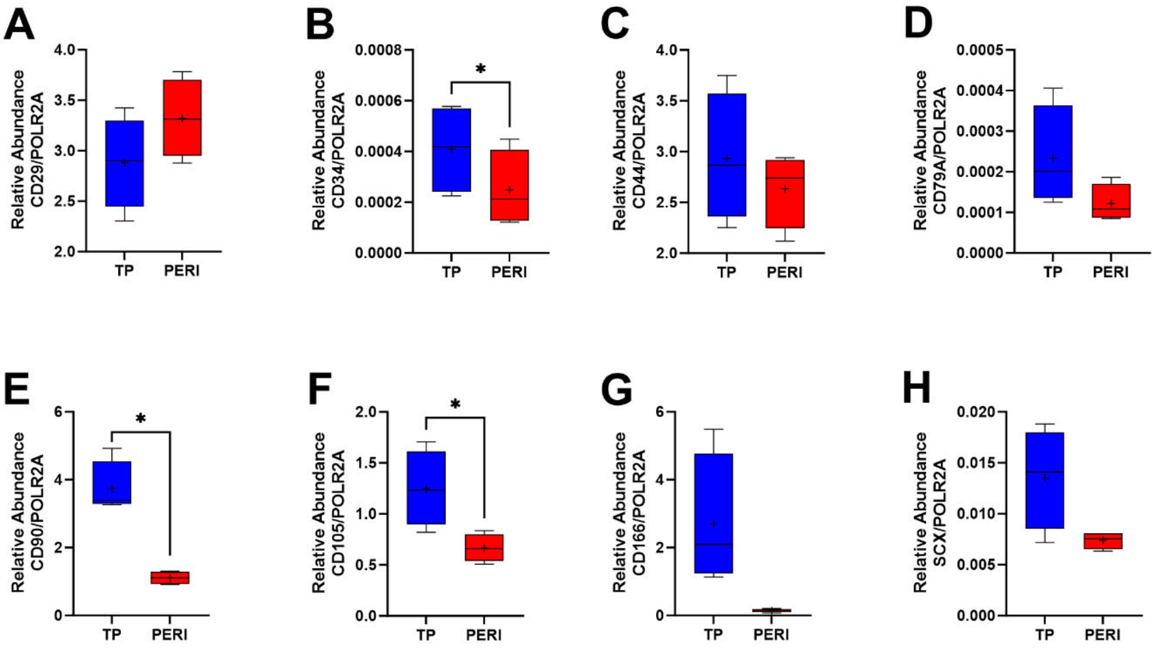

Figure S4. RT-qPCR expression profiles for stem cell markers for TP and PERI progenitor cells. Expression profiles were assayed for TP (blue) and PERI (red) progenitor cells grown in two-dimensional culture after their second passages. Cell markers included: *CD29* **(A)**, *CD34* **(B)**, *CD44* **(C)**, *CD45* (not shown) which had no amplification, *CD79A* **(D)**, *CD90* **(E)**, *CD105* **(F)**, *CD166* **(G)**, and tendon marker *SCX* **(H)**. RT-qPCR statistical analyses performed to examine expression of tendon markers were two-tailed paired t tests (n=4). Significance is demonstrated as *, $p < 0.05$.
